# Fostering medical students’ lifelong learning skills with a dashboard, coaching and learning planning

**DOI:** 10.1007/s40037-018-0449-2

**Published:** 2018-09-05

**Authors:** Karen E. Hauer, Nicholas Iverson, Alekist Quach, Patrick Yuan, Stephanie Kaner, Christy Boscardin

**Affiliations:** University of California, San Francisco, CA USA

**Keywords:** Lifelong learning, Dashboard, Coaching, Clinical competence

## Abstract

**Introduction:**

To develop lifelong learning skills, students need feedback, access to performance data, and coaching. A new medical curriculum incorporated infrastructural supports based on self-regulated learning theory and the Master Adaptive Learner framework to engage students in reflection and learning planning. This study examines students’ experience with a performance dashboard, longitudinal coaching, and structured time for goal-setting.

**Methods:**

Focus groups with first-year medical students explored performance dashboard usage, coaching and learning planning. We analyzed findings using thematic analysis. Results informed development of a 29-item survey rated strongly disagree (1) to strongly agree (5) to investigate experience with the dashboard, coaching and learning goals program. The survey was distributed to one first-year medical student class. We performed descriptive statistics and factor analysis.

**Results:**

In three focus groups with 21 participants, students endorsed using the dashboard to access performance information but had trouble interpreting and integrating information. They valued coaches as sources of advice but varied in their perceptions of the value of discussing learning planning. Of 152 students, 114 (75%) completed the survey. Exploratory factor analysis yielded 5 factors explaining 57% of the variance: learning goals development (α = 0.88; mean 3.25 (standard deviation 0.91)), dashboard usage (α = 0.82; 3.36 (0.64)), coaching (α = 0.71; 3.72 (0.64)), employment of learning strategies (α = 0.81; 3.67 (0.79)), and reflection (α = 0.63; 3.68 (0.64)).

**Discussion:**

The student performance dashboard provides efficient feedback access, yet students’ use of this information to guide learning is variable. These results can inform other programs seeking to foster lifelong learning skills.

**Electronic supplementary material:**

The online version of this article (10.1007/s40037-018-0449-2) contains supplementary material, which is available to authorized users.

## What this paper adds

To develop lifelong learning skills, students need feedback, access to performance data, and coaching. Curricular strategies to support the development of these skills are lacking. Informed by self-regulated learning theory and the Master Adaptive Learner framework, a new medical school curriculum incorporated infrastructure supports with an innovative electronic performance dashboard, longitudinal coaching, and structured time for goal setting. Results of this study using focus groups and a survey aligned with the Master Adaptive Learner framework show that students identify some value of aspects of lifelong learning but do not engage in a cycle of planning, monitoring and adjusting their learning.

## Introduction

Lifelong learning behaviours essential for physician practice entail personal development through continuous acquisition of knowledge and skills, with focus on learning, adapting and discovering [1–3]. To guide students in cultivating habits of continuous improvement for integration into practice, educators must encourage practice, reflection and reinforcement [4]. This approach to learning can improve performance and enhance a learning climate [5, 6]. However, medical school curricula do not typically provide early learners with the support and opportunity to engage in new approaches to learning, assess the impact of these approaches, and reattempt under the guidance of experienced mentors.

The Master Adaptive Learner conceptual framework describes how students can engage in reflection on performance to promote habits of continuous learning and self-improvement [7]. The Master Adaptive Learner purposefully plans for learning, uses intentional learning strategies, self-assesses progress, and adjusts in repeated cycles of learning and adaptation [7]. These steps align with the tenets of self-regulated learning theory [8]. Provision of timely, accurate information about a learner’s own performance enables ‘informed self-assessment,’ which is more accurate than self-assessment unguided by feedback [9].

Critical to student success as a Master Adaptive Learner is adequate infrastructural support to operationalize four steps: planning, learning, assessing and adjusting [7]. Educators must provide learners with information, resources and support for adequate reflection for meaningful self-improvement. Students’ reflections on their performance, including strengths and areas for improvement, should be informed by comprehensive qualitative and quantitative data [9–11]. As such, educational dashboards are now being adopted by some training programs to make performance data readily available [12]. To undergo self-improvement via dashboard review, students actively interpret information through steps that align with the Master Adaptive Learner: awareness and visualization of data; self-reflection and data interpretation; sense-making to construct goals and learning plans; and adjusting performance via behavioural change [13]. Engaging in informed self-reflection with a longitudinal coach enhances students’ ability to create useful learning goals for continuous improvement [14].

Informed by the Master Adaptive Learner framework, a new medical school curriculum integrated three targeted infrastructural supports: an individual performance dashboard, coaching relationship and opportunities to reflect on performance and set learning goals. This study explores how students engage in self-regulated learning behaviours with these infrastructural supports and how they value each support. Findings may inform educators about engaging early medical learners in the behaviours of lifelong learners.

## Methods

### Design

This mixed methods study uses an exploratory sequential design [15]. Mixed methods are appropriate when data collected with one method inform additional data collection with another method, thereby expanding the range of information [16]. Qualitative focus groups revealed medical students’ descriptions and perceptions about the various infrastructural supports designed to facilitate self-regulated learning behaviours and informed design of a quantitative survey of students’ engagement in these learning behaviours. The survey purpose was to quantify students’ priorities and values related to multiple infrastructural supports to guide the school on further interventions including faculty development.

### Setting

The University of California, San Francisco, (UCSF) School of Medicine is an urban, public research-intensive institution in California, USA. In fall 2016, the School launched its new Bridges curriculum, which emphasizes health systems science, inquiry, and longitudinal foundational and clinical sciences learning. The School’s competencies and milestones guide curriculum and assessment [17] (https://meded.ucsf.edu/md-program/current-students/curriculum/md-competencies) as part of programmatic assessment [18]. Three infrastructural supports promote lifelong learning skills in the new curriculum:

#### Dashboard:

An electronic learner performance dashboard displays quantitative and qualitative data from student performance in various formative and summative assessments [12]. Informed by general and targeted needs assessments by the school’s educators and a literature review, the dashboard centralizes and displays all assessment and performance data in a timely manner with performance metrics for easy interpretation with benchmark data and standards [19, 20]. Student dashboard access is limited to the student, coach, deans, and staff who maintain the dashboard.

#### Coach:

Each student has a faculty coach for guidance and support throughout medical school; coaches support approximately six students each in two medical school cohorts. Students meet with their coaches four times in the first phase (18 months) of the curriculum for individual performance reviews and goal setting, and weekly for clinical skills learning. Coaches receive training to use the dashboard and undergo simulation training on development of learning goals and mentorship through standardized student encounters.

#### Reflection and goal-setting:

Students are introduced to the cycle of self-regulated learning early in the curriculum. They learn that their dashboard is a tool to use both independently and with their coach to monitor their progress and create SMART (Specific, Measurable, Attainable, Result-Based, and Time-Bound) learning goals [21]. Assessment-Reflection-Coaching-Health (ARCH) weeks provide structured times for students to reflect and meet with coaches. Students and coaches are advised to gauge students’ performance on the school’s milestones relative to expectations and to peers, and to view students’ prior individual learning goals in the dashboard.

The UCSF Institutional Review Board approved the study. The work was carried out in accordance with the Declaration of Helsinki.

### Phase 1: Qualitative

#### Subjects and sampling:

We used convenience sampling for focus groups. First-year students were invited through the class listserve to participate. All respondents to a first email who were able to attend a scheduled focus group were included.

#### Data collection:

In spring 2017, two trained moderators, a fourth-year medical student (AQ) and research assistant (PY), led three 50-minute focus groups. Students provided written informed consent and completed a five-item demographic survey. Focus group questions explored students’ understanding of the purpose of the electronic dashboard and how and why they used it, their use of other sources of performance information, and their coach interactions to review performance and create learning plans (see Appendix 1 in the Online Electronic Supplementary Material). All focus groups were audiotaped, professionally transcribed, and de-identified.

#### Analysis:

Data analysis occurred concurrently with data collection for early analysis to iteratively inform subsequent data collection. Three investigators (a fourth-year medical student, research assistant, and faculty member (AQ, PY, KEH)) analyzed focus group data using thematic analysis. As data were collected, these three investigators read each transcript independently to identify key themes. Through iterative discussion, they refined the themes into a codebook. Using the constant comparative method, they compared findings within and across transcripts [22]. Two investigators independently coded the transcripts and reconciled discrepancies through discussion. These three investigators reviewed the coded data to identify relationships among codes and synthesize information into larger themes. Data collection continued until sufficient information was obtained about the infrastructural supports to inform survey design. Investigators used Dedoose software, version 6.1.18 (SocioCultural Research Consultants, LLC, Los Angeles, California) to code, organize and retrieve coded data.

Investigators considered reflexivity throughout data collection and analysis [23] by repeatedly sharing with one another their perspectives based on their experience and role in the school and how this influenced their reactions to the data.

### Phase 2: Quantitative

#### Subjects and sampling:

All 152 finishing first-year students received an email invitation for the survey through Qualtrics (Provo, UT) in April 2017. Non-responders received up to two email reminders.

#### Data collection:

Informed by focus group results and the Master Adaptive Learner conceptual framework [7], three investigators (KEH, NI, CB) developed draft survey items following procedures for survey design [24]. Items targeted students’ experience with and perceptions of the utility of new curricular infrastructures designed to facilitate self-regulated learning behaviours. Focus groups and the Master Adaptive Learner framework prompted item generation addressing dashboard use alone and in coaching encounters, discussing performance, informed-self assessment, reflection, and goal-setting. Investigators initially drafted 31 items that each mapped to a phase of the Master Adaptive Learner framework. A14-member health professions education panel of clinicians, statisticians, and medical students reviewed draft survey items. Based on their feedback, two irrelevant or redundant items were removed. The final 29 items addressed the four phases of the Master Adaptive Learner framework: Planning (5), Learning (7), Assessing (12), and Adjusting (5). Respondents rated their level of agreement for each item on a 5-point Likert-type scale (1 = strongly disagree, 5 = strongly agree).

#### Analysis:

Responses were de-identified before analysis. We calculated descriptive statistics for all 29 items. Exploratory factor analysis with varimax rotation identified latent variables characterizing students’ perceptions of the value of the resources and activities related to the Master Adaptive Learner framework. Although latent variables may not be readily apparent, they can drive responses to survey items [25]. Factors with eigen values greater than 1 were retained. Items with factor loadings of 0.4 were considered significant for this sample size [26]. Cronbach’s α, mean and standard deviation (SD) were calculated for each factor. We compared composite means by conducting *t-tests* with Bonferroni correction (0.05/10 = 0.005) for multiple comparisons to avoid finding significant results by chance. We used SPSS for Windows Version 24.0 for quantitative analyses (IBM SPSS Statistics for Windows, Version 24.0. Armonk, NY: IBM Corp.)

## Results

### Phase 1: Qualitative

Twenty-one students (14 women, 7 men; mean age: 24 years, range 21–31) participated in three focus groups (3, 8, and 10 participants). Nine (45%) had previous experience creating learning goals, in earlier education or work. Focus group results addressed three themes: information access, information interpretation, and coaching for change.

#### Information access:

Most students described using the dashboard primarily to view score reports from written examinations and other performance reports that were automatically uploaded. One student explained: *‘I think of it as the assessment help, and for any type of assessment I can find out how I did there.’ *(Group 2) While students in each group initially described obtaining score reports in the dashboard, each group then characterized other purposes of the dashboard, including capturing individual and longitudinal performance information. One student noted: *‘It’s one of the few sites where I feel like it’s personal to me.’ *(Group 3)

#### Information interpretation:

Students had difficulty interpreting all information housed in the dashboard independently. Viewing information about their performance seemed to provide them some information while also raising many questions. One student observed: *‘It’s still a little bit vague, but that’s the information we have on how we’re doing.*’ (Group 3) They desired more feedback, more longitudinal performance views, transparency in grading, and individualized comments on all assessments. Many students shared technical questions about particular dashboard features or suggested features that could be added. Many wanted more detailed information about their performance particularly relative to peers. The dashboard raised concerns for some about how the school might use performance information in ways they might not know to compare them with peers and school expectations.

#### Coaching for change:

Students praised their coaches highly as supports and mentors, yet expressed mixed opinions of their experience discussing learning planning with coaches. For some students, the process of setting learning goals for posting in the dashboard and discussing with the coach seemed productive. Coach discussions enhanced students’ perceptions of their ability to interpret their own progress:

*I’ve actually found it really helpful to have time one on one to talk about academic things and SMART goals because I feel like sometimes the feedback from tests is super generic. You either met expectations, you were borderline, or you didn’t, and I often don’t really know what does that mean for what I’m doing and what I need to change?* (Group 3)

In required progress meetings with coaches, some students viewed the dashboard together with coaches and found this step enlightening. One student appreciated that the coach advised: *‘See, by itself, this may be not the most useful thing in the world, but over time you can see a trend.’* (Group 1) Another student described the coach providing clarification about information in the dashboard: *‘With her eye, she can also make sense of the information and tell me so this is how it can be helpful for you.’* (Group 1).

Students perceived learning plan development with coaches to be more effective when goals were meaningful to the student rather than seeming to be dictated by data in the dashboard. They appreciated longitudinal coach relationships for individualized guidance and reassurance. They shared tentative thoughts that they might be developing a habit of mind through reflecting and articulating goals. One student characterized progress meetings: *‘By giving us this time almost off they’re forcing us to zoom out and step back and say, hey, wait, what am I working towards?’* (Group 1).

However, others reported that coaches did not steer them to use the dashboard together; those students trusted that their coaches had checked the dashboard in advance to ensure performance met expectations. Students valued agenda flexibility to address issues most salient to the student when meeting with coaches. Students reported that coaching meetings focused on varying topics including general academic support, career guidance, and students’ personal wellbeing:

*I think coaching time is to just talk about what you want. People in our sessions have talked about very personal issues, people have talked about the curriculum, people have talked about SMART goals …just that openness … that’s a really helpful setting*. (Group 2)

Multiple students struggled with figuring out how creating learning goals would benefit them. Some felt they already used effective learning strategies:

*It’s not helpful to my learning or my long-term career goals because often what I’m doing is taking things that I’m already doing and then somehow squishing them into this format that I can just put on this dashboard. *(Group 1)

Another barrier was the perception that completed learning experiences cannot be changed: *‘The dashboard is post-exam; it’s like, Well, there’s nothing I can do about that now.’ *(Group 3).

Some students were still considering how to engage in the process of using performance review to identify and implement goals. One student explained:

*I don’t always go into meetings with my coach with clear hopes or goals, but actually for my last session I had no ideas, and she was really helpful in talking to me and figuring out where to improve.’ *(Group 3)

Many students similarly described reviewing their performance without having previously engaged in planning as part of a cyclic process.

### Phase 2: Quantitative

Overall, 114 of 152 first-year students completed the survey (75%). Tab. 1 provides survey responses.

**Table 1:** 114 first-year medical student responses to a survey on dashboard usage, coaching and learning planning, University of California, San Francisco, School of Medicine

Factors (α coefficient)	Item	Mean^a^	Standard deviation (SD)	Factor mean (SD)
Factor 1: SMART^b^ goals (α = 0.88)	I regularly set SMART goals for myself to improve my learning	2.82	0.99	3.25 (0.91)
	SMART goals are a useful tool	3.36	1.02	
	I find it useful to create SMART goals related to my academic performance	3.01	1.15	
	I find it useful to create SMART goals related to my career planning	3.49	1.15	
	I find it useful to create SMART goals related to my well-being	3.75	1.01	
	I keep track of the progress of my SMART goals	2.97	1.02	
Factor 2: Dashboard usage (α = 0.82)	I usually choose which goal to create based on data on my performance from the dashboard	2.45	1.14	3.36 (0.64)
	I am able to review my individual performance readily using the dashboard	3.67	0.94	
	It is helpful to review my performance compared with other students using the dashboard	3.71	0.97	
	The breadth of data in the dashboard is sufficient for me to assess my academic performance	3.32	0.94	
	The exam grade reports in the dashboard help in assessing my performance	3.55	0.90	
	I find it easy to track my exam scores over time to monitor improvement in my performance	3.25	0.96	
	Reviewing my dashboard motivates me to improve my performance	3.54	0.87	
	The information about my performance in the dashboard is accurate	3.70	0.78	
	The information about my performance in the dashboard reflects my progression as a medical student	3.40	0.82	
Factor 3: Learning strategy (α = 0.81)	I use effective learning tools and strategies	3.91	0.68	3.72 (0.64)
	When I study, I test myself to check my understanding of what I’ve studied	3.94	0.86	
	I know how to identify the best learning resources to make progress in an area that I need to improve in academically	3.46	0.91	
	I have the skills required to be a lifelong learner	4.08	0.75	
	I am adequately supported to implement my SMART learning goals	3.68	0.73	
	I currently know how to interpret feedback and data/scores to improve my academic performance	3.48	0.81	
Factor 4: Coaching for improvement (α = 0.71)	My coach helps me create or adjust my SMART goals	3.83	0.88	3.67 (0.79)
	Working with my coach helps me understand how to use performance data and feedback to create SMART learning goals	3.52	1.02	
	My coach helps me to assess and reflect on the progress I have made on my goals	3.80	0.84	
Factor 5: Reflection (α = 0.70)	When I receive feedback, I often reflect and set new goals	3.79	0.81	3.68 (0.64)
	I direct my learning based on my individual learning needs	4.19	0.72	
	I mainly use sources other than the dashboard to review how I am doing in medical school	2.94	0.97	
	If I’m not on track with my goals, I try to make adjustments and find resources to achieve those goals on my own	3.92	0.80	
	When I receive feedback, I use that information to change my behaviour	4.18	0.59	

^a^(1–5, 1 = strongly disagree, 5 = strongly agree)

^b^SMART (Specific, Measurable, Attainable, Result-Based, and Time-Bound)

Factor analysis yielded five factors explaining 57% of the variance characterizing students’ perception of the various Master Adaptive Learner behaviours and infrastructure components. Cronbach’s alpha for the five factors ranged from 0.70 to 0.88. Factor 1 represented items related to *learning goals development* (6 items, factor mean 3.25, SD 0.91). Factor 2 addressed *dashboard usage* (9 items, mean 3.36, SD 0.64). The third factor addressed employment of *learning strategies* (6 items, mean 3.72, SD 0.64). The fourth factor focused on *coaching* (5 items, mean 3.67, SD 0.79). The fifth factor addressed *reflection* (5 items, mean 3.68, SD 0.64). Students’ perception of learning strategies (*p* < 0.000), coaching (*p* < 0.000), and reflection (*p* < 0.000) were all significantly higher compared with dashboard usage and learning goals development. Three individual items with the most positive responses (mean >4) pertained to students’ endorsement of their own skills in self-directed and lifelong learning, and incorporation of feedback.

## Discussion

Guided by the Master Adaptive Learner framework, the new curriculum focused on three targeted infrastructural supports: individual performance dashboard, coaching relationship, and opportunities for reflection and goal-setting. This mixed methods study illustrates how students vary in their understanding of connections between performance information, reflection and working with coaches to enact personal change. Their survey results showed mild endorsement of the value of infrastructural supports for self-regulated learning behaviours, with more positive perceptions of their own learning strategies, coaching for improvement and their own reflection than for learning goals development and dashboard usage. Findings suggest that students seem to need more training, coaching, support or experience to develop all Master Adaptive Learner skills. Similarly, a recent study of resident learning goals showed that residents struggled with lifelong learning behaviours and required heavy faculty support to use learning goals [27].

Despite experiencing SMART goals and a performance dashboard within the curriculum structure, most students did not report optimally utilizing these to engage in new or different approaches to their learning. Development of lifelong learning skills requires practice with specific coaching [4]. Improved orientation to students could enhance their understanding of the cyclical process of self-regulated learning as described in the Master Adaptive Learner framework and the potential utility of infrastructure supports to actualize lifelong learning skills [28]. As our participants progress into clerkships, the perceived value of information about their workplace performance could become a more prominent driver for continuous enhancement [29]. Our students’ perceptions of themselves as skilled lifelong learners contradicts their reported engagement with the tools and activities of lifelong learners. Other literature corroborates the limitations of self-assessment of one’s skills [30]. Our participants’ emphasis on the value of coaching reinforces this essential ingredient for enhancing the usefulness of assessment activities for promoting learning [31, 32].

Focus group participants valued dashboard information as evidence of their achievements which may suggest a performance rather than a mastery perspective [33]. Students sought dashboard content for comparison with peers and assessment results rather than to guide self-improvement. While reinforcement of successful performance can feel validating and potentially enhance performance [5], identification of areas needing growth is essential for physicians to develop competence. Student’s self-efficacy—perceptions of capability to achieve goals—is fostered by enacting adaptive, self-regulated learning behaviours that motivate attempts at new learning activities [34]. Accurate self-assessment achieved through guided feedback focuses self-regulated learning appropriately on tasks for growth [35]. Increased emphasis on formative assessment may facilitate culture change towards mastery, a more adaptive learning approach. This study has limitations. Findings from this single institution study may not generalize to other institutions or later years in medical school. Focus group volunteers may not have represented the whole class. We did not collect data about survey non-respondents, although we did have a high survey response rate. We did not measure learning strategies or include performance measures to determine the effects of particular strategies on learning goal accomplishment. This study demonstrates that early medical students view themselves as skilled with learning strategies yet use a performance dashboard primarily to confirm performance achievements and find lower value in setting learning goals. Additional practice and support may enable students to link pieces of the Master Adaptive Learner cycle to instil habits of lifelong learning.

## Caption Electronic Supplementary Material

Appendix 1. Focus Group Facilitator Script
