# Supplementary material for: Fostering medical students’ lifelong learning skills with a dashboard, coaching and learning planning
Source: Perspect Med Educ. 2018 Sep 5;7(5):311–7. doi: 10.1007/s40037-018-0449-2 (PMC6191394; doi:10.1007/s40037-018-0449-2)
Supplement: Supplementary file 1 — Appendix 1. Focus Group Facilitator Script [file 40037_2018_449_MOESM1_ESM.docx]

**Appendix 1. Focus Group Facilitator Script**

*Introduction to Focus Group: Thank you for your willingness to participate in this focus group. This focus group is one of many conducted as part of UCSF Bridges curriculum initiatives and is part of a research study about the Bridges Student Dashboard. In this focus group, we hope to discuss your experiences with using the Bridges Student Dashboard and discussing learning goals with your coach.*

*Focus Group Guidelines: We will follow some guidelines during this focus group. What you say will be strictly confidential and will be presented in a summarized format. Your name will not be associated with what you have said, and we ask that students in this group also keep what their classmates have said in confidence. To create an open and honest forum of ideas, we ask that you respect each other’s ideas. We are eager to hear your personal experiences and would prefer that you focus on your own experiences rather than what you may have heard from classmates or peers. We will want to hear about the positives and areas for improvement. If you are unhappy with an experience, please provide suggestions for how those areas could have been designed or executed to better meet your learning needs.*

*Your participation is voluntary. If you agree to these guidelines, please say yes.*

*Introduction to facilitator and his or her role: My role as a facilitator is to listen to your perspectives, balance the conversation, and ensure that the views of all participants in this group are captured and represented. We will use qualitative methods to analyze the data from this group in a report to the UCSF educational leadership, which may effect change in the next iteration of the curriculum or program.*

**A. Tell me about how you use the electronic student dashboard**

1. *How often do you look at it?*
2. *What prompts you to use the dashboard?*
3. *What sections do you look at?*
4. *What information are you seeking?*
5. *What questions are you hoping to answer?*
6. *How do you use the information in the dashboard?*
7. *Does information you see in the dashboard change what you do? Can you give an example?*

**B. What other resources or information do you use to tell you how you are doing in medical school?**

1. *What information or guidance do other resources provide that the medical student dashboard does not provide?*

**C. Let’s talk about your meeting with your coach to go over learning plans.**

1. *How did the meeting begin?*
2. *How did the conversation unfold?*
3. *Did your coach help you identify learning goals? If so, how?*
4. *How did your coach use the dashboard to help with your learning planning?*
5. *What kind of guidance or feedback did your coach provide?*
6. *What did you find most helpful about your meeting with your coach?*
7. *What was not helpful about your meeting with your coach?*
8. *Do you find the process of working with your coach useful in the learning planning process?*
